# Causes for an extreme cold condition over Northeast Asia during April 2020

**DOI:** 10.1038/s41598-023-29934-w

**Published:** 2023-02-27

**Authors:** Go-Un Kim, Hyoeun Oh, Yong Sun Kim, Jun-Hyeok Son, Jin-Yong Jeong

**Affiliations:** 1 Korea Institute of Ocean Science and Technology, Busan, South Korea; 2 Ocean Science and Technology School, Korea Maritime and Ocean University, Busan, South Korea; 3 Korea Power Exchange, Naju, South Korea

**Keywords:** Atmospheric science, Climate change, Environmental sciences

## Abstract

Although 2020 was the fourth warmest year on record in northern Asia, the cold condition in April 2020 caused severe damage to the agricultural and marine ecosystems in northeastern Asia. Previous studies have indicated that the dipole atmospheric circulation over Siberia and the East Sea (Japan Sea) produced this cold environment with strong northwesterly wind. However, the potential causes of the dipole circulation over northeastern Asia remain unclear. In this study, we found that the East Atlantic/Western Russia (EAWR) pattern and blocking combined to produce the atmospheric structure. The wave train originated from the vorticity forcing of northwestern/central Russia and propagated from Western Europe to the East Sea via the background westerly and northerly winds. In addition, the Siberian blocking days increased eleven times in April 2020 relative to the climatological average, and an easterly (westerly) anomaly was observed over Mongolia–northeastern China (northern Russia). The strong blocking and EAWR pattern led to the robust atmospheric dipole structure with a prevailing northerly flow in April 2020, thereby causing the extreme cold condition over northeastern Asia. Our results provide novel insights into the cause of extreme cold condition in April over northeastern Asia.

## Introduction

Humans are exposed to multiple risks under climate change. Extreme weather or climate anomalies can alter the structure of land and ocean ecosystems, as well as species ranges and timing^1^. Ultimately, ecosystem changes adversely impact food production in agriculture, aquaculture, and fisheries^1^. According to previous studies, the hazards have increased through two or more extreme events co-occurring, an extreme event with amplified existing climate conditions, or a combination of events leading to extreme events^2–5^. Thus, it is essential to understand the process and cause of unusual weather/climate phenomena to reduce the loss of socio-economic and human activities.

**Figure 1:**
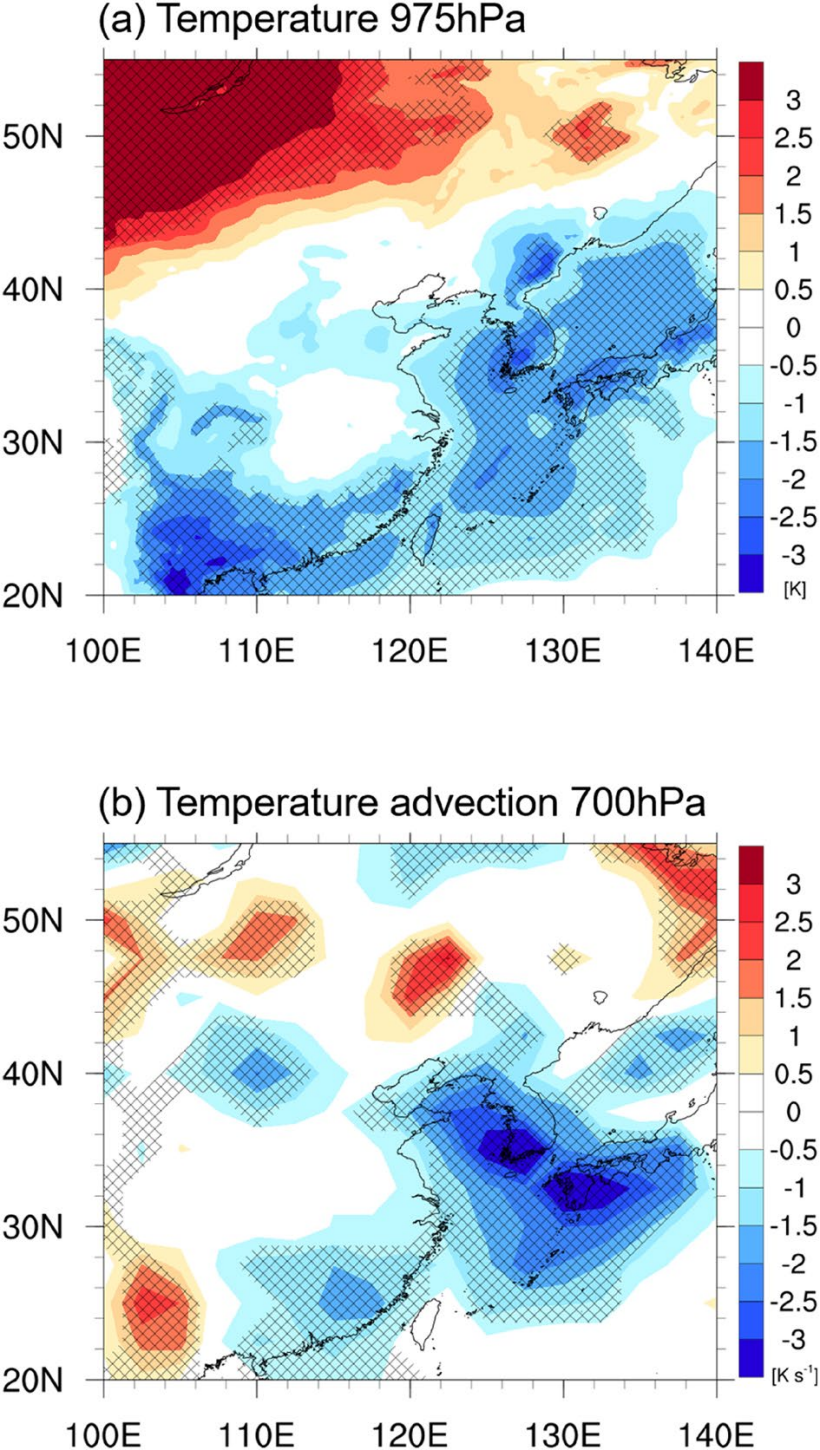
Maps of cold condition in April 2020. Spatial patterns of the (**a**) 975-hPa temperature (shading, K) and (**b**) 700-hPa temperature advection anomalies (shading, 10^−5^ K s^−1^) in April 2020 for ERA5. Hatched areas denote the statistically significant regions at the 90% confidence level. The maps were generated using the NCAR Command Language Version 6.4.0 (http://www.ncl.ucar.edu).

In April 2020, Asia experienced a heterogeneous and extreme temperature distribution from region to region. The temperature in North Asia (Ural and Siberia) was the fourth highest since 1880, while low temperatures were concurrently recorded in Northeast Asia (North China and Korea) associated with strong cold advection (www.ncdc.noaa.gov/sotc/global/202004; Fig. 1). This cold condition over Northeast Asia lasted for approximately the month of April, with ~ 2 days of short-term and ~ 8 days of long-term cold surges (Supplementary Fig. S1). The Ministry of Emergency Management of China considered the extreme cold surge at the end of April as one of China’s top ten natural disasters in 2020. The severe low-temperature event (exceeded −5 °C and decreased below −7 °C in daily mean temperature anomalies)^6^ damaged 530,000 ha of agricultural produce and negatively affected the lives of four million residents in North and Northwest China, with a total economic loss of 1.2 billion dollars (www.cma.gov.cn/2011xwzx/2011xmtjj/202101/t20210104_569543.html). In addition, the extreme cold-related sleet on 22nd April represented late snowfall in Seoul, South Korea, since record-keeping began in 1907. The Ministry of Agriculture, Food and Rural Affairs of Korea reported that the low-temperature event caused spring frost damage to crops on 48,000 ha, hence the government spent 80 million dollars on recovery efforts (www.mafra.go.kr).

The severe cold environment in April 2020 had a negative influence on not only the land in North China and Korea but also the marine ecosystem of the Yellow Sea. Kim et al.^7^ identified the slowest water temperature evolution over the northeastern basin of the Yellow Sea for spring 2020 in the last four decades. This record-breaking evolution from March to May was principally attributed to the exceptional latent heat release in April 2020 via robust northwesterly wind and high sea surface temperature that was partially caused by warm winter water. The considerable heat loss from the ocean to the atmosphere generated the cold-water anomaly on the sea surface from April to May, while a warm anomaly in winter persisted below the thermocline even during spring. The subsequently weakened stratification could impede the increased chlorophyll-a concentration in the euphotic layer. This unfavorable condition for phytoplankton activity likely affected the delayed and suppressed spring bloom in 2020. The satellite-derived phytoplankton biomass decreased by ~ 30% over the Yellow Sea in April 2020 compared with that in the normal years from 2015 to 2019^8^.

The prevailing northerly wind with a cold air mass over Northeast Asia tends to occur during winter^9–13^. The amplification of the Siberian High mainly explains the cold winter conditions through the upper-tropospheric Rossby wave train and blocking processes^9–13^. For the stationary Rossby wave dynamics, atmospheric waves induced by upper-tropospheric disturbance propagate across Eurasia. These waves amplify the downstream anticyclonic and cyclonic anomaly circulations through interactions with the pre-existing Siberian high, thereby facilitating the invasion of northerly cold air in East Asia^9,11–13^. The blocking pattern process interacts with the complex orography and large-scale circulation variability, such as Arctic Oscillation (AO), thereby reinforcing and expanding the Siberian High and promoting the movement of cold air masses to East Asia^10–13^.

Interestingly, such a cold winter environment occurred during April 2020 in Northeast Asia. Previous studies have proposed that the anomalous anticyclonic circulation was located over Siberia, accompanying the cyclonic circulation anomaly over the East Sea (also referred to as the Japan Sea) in April 2020^6,7^. This dipole circulation led to the strong northerly flow resulting in the cold condition in Northeast Asia^6,7^. However, the specific cause of the atmospheric dipole structure in April 2020 remains unknown. Therefore, we examine the characteristics and potential causes of the large-scale atmospheric circulation related to the extremely cold condition in April over northeastern Asia.

## Results

### Characteristics of the atmospheric circulation in April 2020

**Figure 2:**
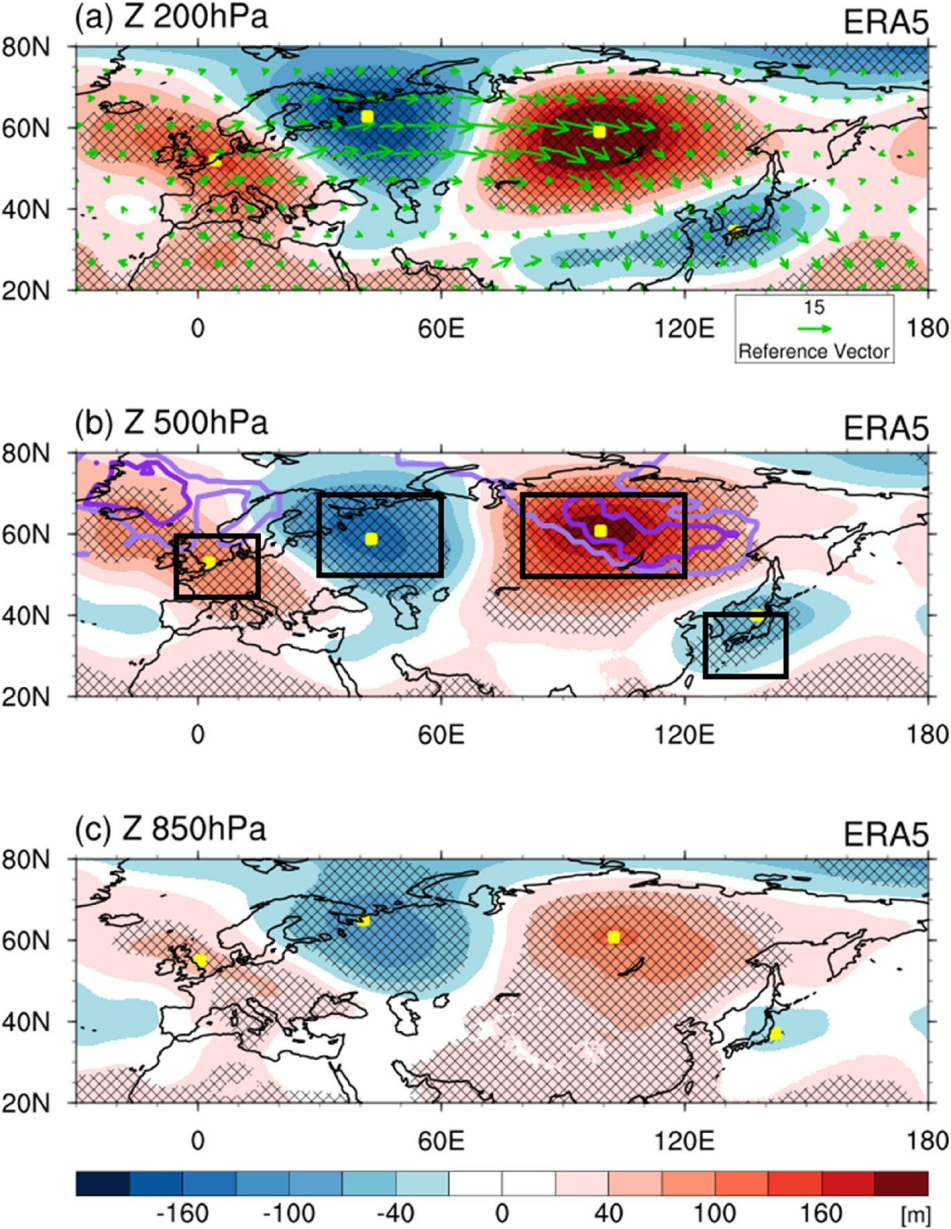
Characteristics of atmospheric circulation anomalies in April 2020. Spatial maps of the (**a**) 200 hPa, (**b**) 500 hPa, and (**c**) 850 hPa geopotential height (shading, m), 200 hPa wave activity flux (vector, m^2^ s^−2^), and blocking frequency (contour, days per month) anomalies in April 2020 for ERA5. Yellow squares indicate the location where the maximum or minimum geopotential height anomalies. Hatched areas denote the statistically significant regions at the 90% confidence level. In (**a**), the reference wave activity flux vector is 15 m^2^ s^−2^. In (**b**), the light and dark purple contours indicate the area with blocking days of more than 10- and 15-days per month in April 2020 compared with the climatological average. Black boxes in (**b**) represent the regional domains information for the three indices from left to right: Western/Central Europe (45–60°N, 5°W–15°E), northwestern/central Russia (50–70°N, 30–60°E), Siberia (50–70°N, 80–120°E), and the East Sea (25–40°N, 125–145°E). Maps were created using the NCAR Command Language Version 6.4.0 (http://www.ncl.ucar.edu).

Figure 2 illustrates the 200, 500, and 850 hPa geopotential heights, 200 hPa wave activity flux, and blocking frequency anomalies for April 2020 using the European Centre for Medium-Range Weather Forecasts Re-analysis v5 (ERA5). In the east–west direction, the anticyclonic circulation anomalies appeared over Western/Central Europe (45–60°N, 5°W–15°E) and Siberia (50–70°N, 80–120°E) near Lake Baikal. The cyclonic circulations occurred over northwestern/central Russia (50–70°N, 30–60°E) and the East Sea (25–40°N, 125–145°E) with alternating signs (i.e., in a ridge-trough-ridge-trough pattern). Besides, the location of maximum intensity for the anomalous ridge over Siberia/Russian Far East and the location of minimum intensity for the anomalous trough over the East Sea/Northwest Pacific slightly tilted westward with height in the troposphere, in which the vertical structure indicates the growth of baroclinic disturbance. This atmospheric circulation structure is analogous to a wave train pattern. The wave activity flux was calculated using the method of Takaya and Nakamura^14^ to estimate the propagation path of Rossby waves (Fig. 2a green vectors). The wave-flux vector started from Western Europe and seemed to proceed towards the marginal northwestern Pacific, which coincided with the ridge-trough-ridge-trough pattern.

A series of anticyclonic and cyclonic circulation anomalies exhibited over Northeast Eurasia in the north–south direction. The anomalous ridge over Siberia/Russian far East had a grossly equivalent barotropic vertical structure, but the anomalous trough over the East Sea/northwestern Pacific had an apparent westward tilt of about 10° in height. These barotropic ridge and baroclinic trough systems are comparable to a blocking pattern. To identify the blocking occurrence, the blocking days were estimated using the hybrid atmospheric blocking method^15,16^, which combines each advantage of the Dole–Gordon index^17^ and the Tibaldi–Molteni index^18^. The blocking days over Siberia during April 2020 occurred approximately eleven times more relative to that in the climatological period from 1982 to 2019 (by an area average of 11.6 days in 2020 and 1 day for the climatological average; Supplementary Fig. S2, Fig. 2b, light and dark purple contours). These results suggest that the atmospheric structure associated with the cold condition in April 2020 might be attributed to a mixed type of wave train and blocking.

**Figure 3:**
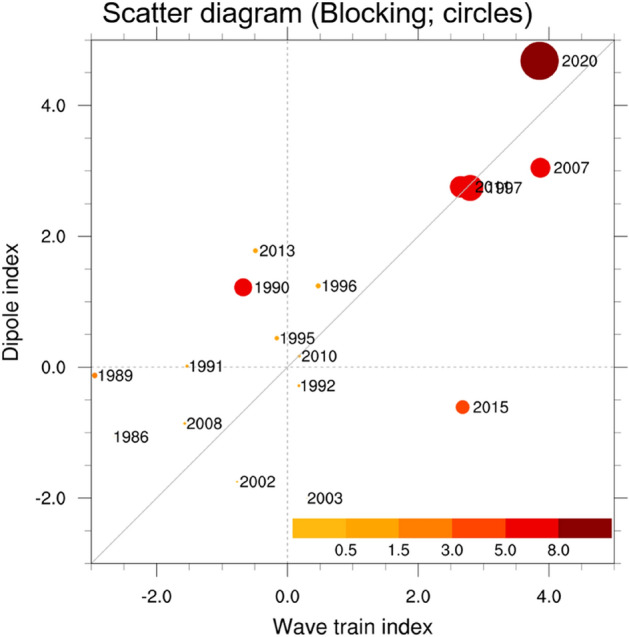
Comparison between April 2020 and normal years on the atmospheric circulation linked to the cold condition. Scatter diagram of the wave train index (*x*-axis) under dipole index (*y*-axis) and blocking index (circles) during April 1982–2020, except for the year with a zero-blocking index (i.e., non-blocking). Each circle’s size and color (from yellow to red) denote the magnitude of the blocking index, indicating the blocking frequency in Siberia. Each year is presented on the right side of the circles.

To determine how different the atmospheric circulation related to the cold condition in April 2020 compared with normal years, we plotted a scatter diagram of the dipole pattern (ordinate) under the wave train (abscissa) and blocking (maker) indices (Fig. 3). The dipole atmospheric circulation index is considered as the cold condition with the northerly flow in Northeast Asia. The wave train and blocking indices are potential causes of cold condition (see indices details in the "Methods" section). Blocking events over Siberia are uncommon during April (Supplementary Fig. S2a), and such events occurred in only 17 of the 39 years from 1982 to 2020. For the 17 years, the dipole circulation index showed a robust linear relationship with the wave train index (correlation coefficient = 0.6, *p* = 0.01). These large wave train and dipole indices are often relevant to the high blocking indices, as observed in the years 1997, 2007, 2011, and 2020. Of note is that the wave train index in April 2020 was nearly the same as that in 2007 (i.e., 3.9), while the dipole index value differed considerably by 4.7 in 2020 and 3 in 2007. Therefore, this larger dipole index in 2020 is relevant to the extreme blocking index of 11.6 in 2020, which was the strongest in 39 years (for example, the blocking index value was 6 in 2007). These results indicate that this exceptional Siberian blocking could play a crucial role in modulating the dipole atmospheric circulation over Siberia and the East Sea in concert with the wave train pattern.

### Possible causes of the cold condition in April 2020

**Figure 4:**
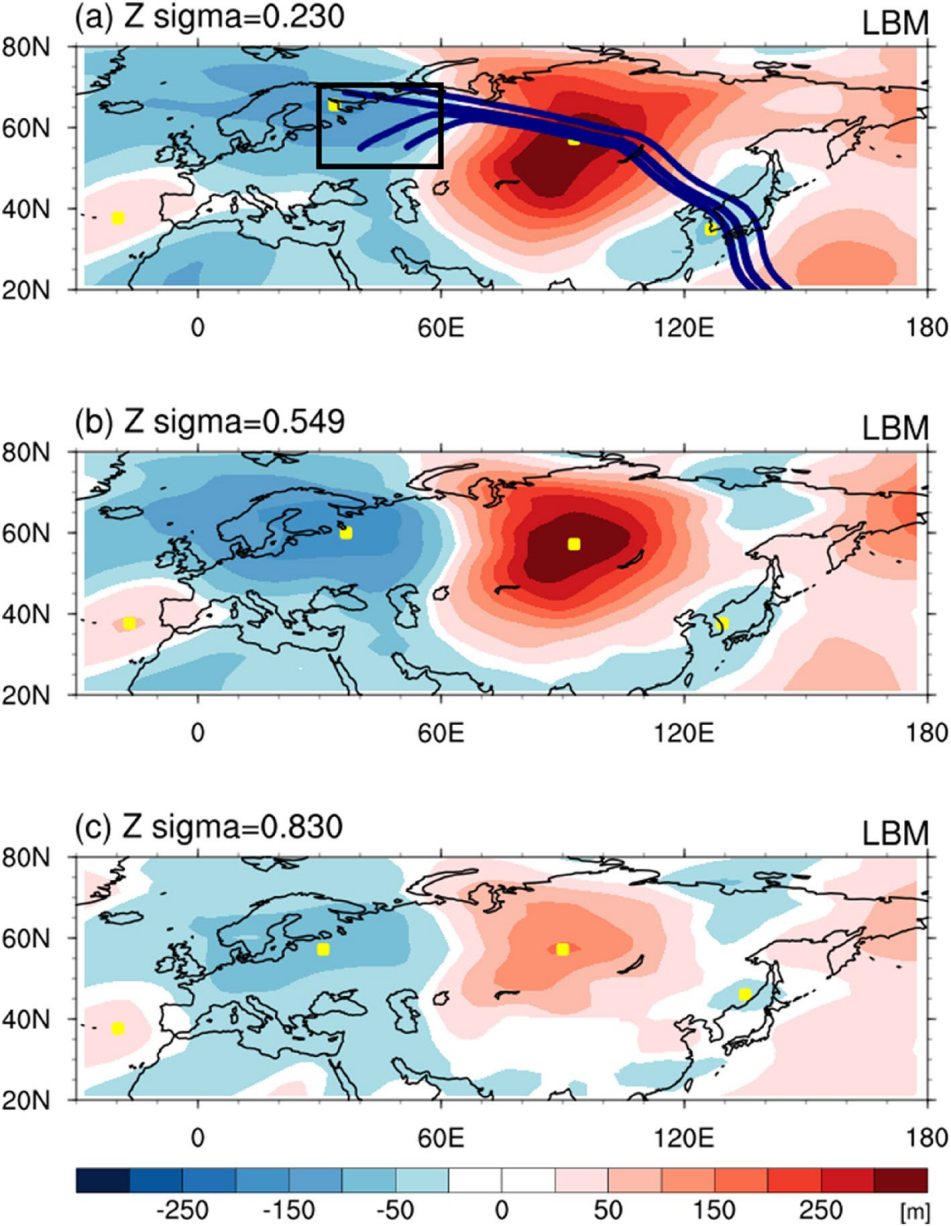
Simulated atmospheric circulation anomalies in response to vorticity forcing. (**a**) 0.230σ, (**b**) 0.549σ, and (**c**) 0.830σ geopotential height anomalies (shading, m) averaged over 16–20 days of output in response to vorticity forcing. In (**a**), navy lines represent the Rossby wave ray path for waves with the zonal wavenumber 2, while the black box indicates the vorticity forcing region (northwestern/central Russia; 50–70°N, 30–60°E). Maps were generated using the NCAR Command Language Version 6.4.0 (http://www.ncl.ucar.edu).

A question arises as to which forcing can form the atmospheric circulation in April 2020 over Eurasia. To this end, we performed a numerical experiment using the linear baroclinic model (LBM) that is capable of diagnosing the steady-state atmospheric dynamical response to prescribed forcing^19–21^. The experiment was forced with the positive vorticity tendency profile for the northwestern/central Russia region obtained from ERA5 (black box in Fig. 4a, Supplementary Fig. S3) based on the results of the wave activity flux and 200 hPa geopotential height anomaly. The simulated upper-, mid-, and low-level geopotential height anomalies for near steady-state (averaged over 16–20 days) are plotted in Fig. 4. All tropospheric geopotential height anomalies for the vorticity forcing exhibited horizontally and meridionally alternating positive and negative signs over Eurasia. The spatial correlation between simulated and observed 200 hPa geopotential height anomalies is 0.71 over 25–80°N and 0–150°E, which is significant at the 99% confidence level. This correlation indicates that the numerical experiment reasonably reproduced the observed atmospheric circulation in April 2020. On the other hand, the location of positive height anomalies in Western Europe was not well simulated in the model compared to the reanalysis (Figs. 2, 4), presumably relating to the diabatic or vorticity forcing in the North Atlantic or tropics^20,22,23^.

**Figure 5:**
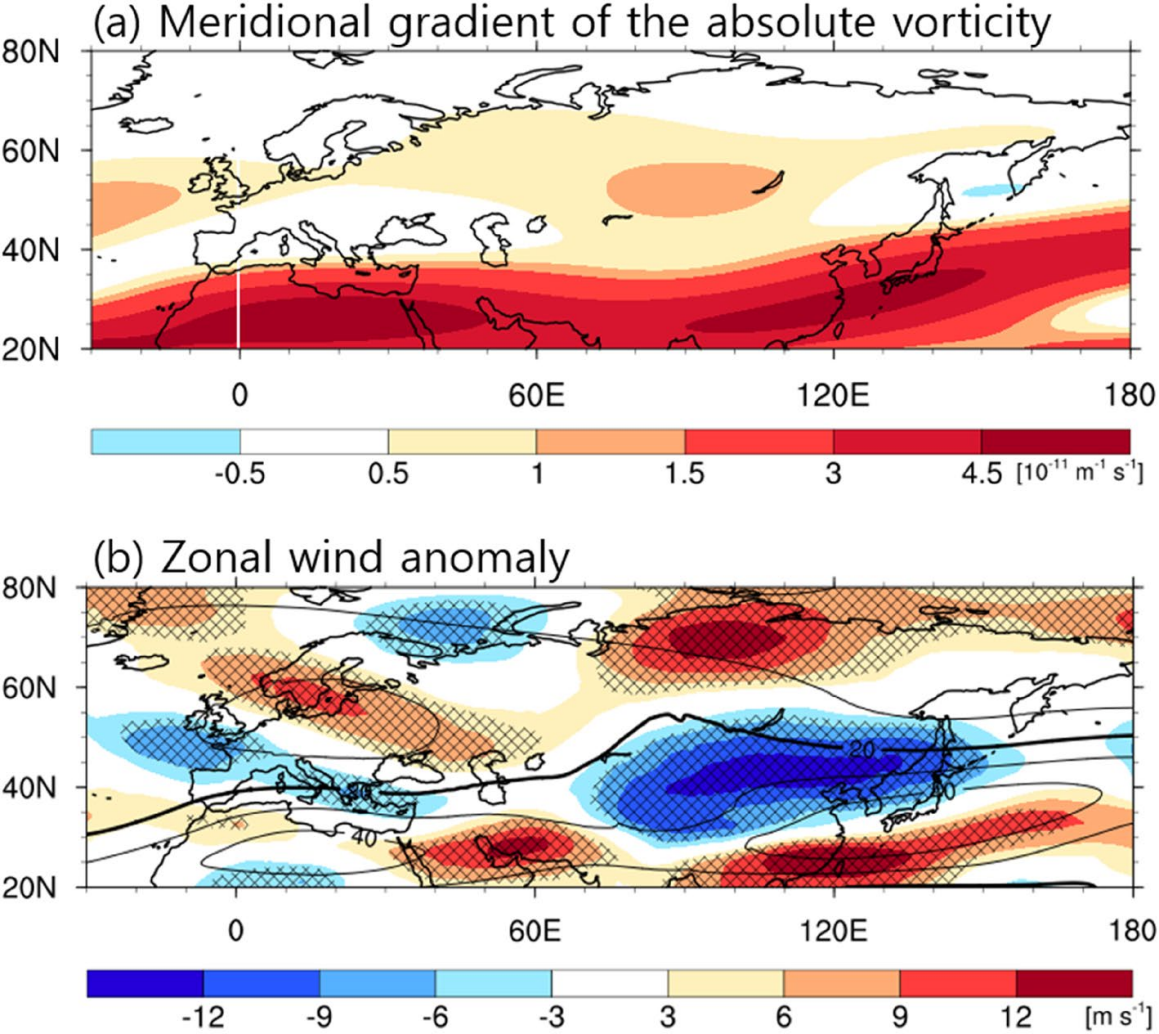
Potential causes for the cold condition. Upper-troposphere (200 hPa) (**a**) meridional gradient of the absolute vorticity (${\overline{q} }_{y}$, 10^−11^ m^−1^ s^−1^) climatology in April at the Mercator coordinate and (**b**) zonal wind (m s^−1^) in April 2020 anomaly (shading) and climatology (contour, interval of 10 m s^−1^). In (**b**), hatched areas represent statistically significant regions at the 90% confidence level. The maps were created using the NCAR Command Language Version 6.4.0 (http://www.ncl.ucar.edu).

The atmospheric circulation anomalies in the horizontal direction have an arc-shaped wave pattern (Fig. 4). The ray tracing method was applied to identify the Rossby wave energy propagation. The calculated rays for zonal wavenumber 2 matched well with the centers of upper-tropospheric geopotential height anomalies (navy lines and yellow squares in Fig. 4a), implying the vorticity forcing at northwestern/central Russia could generate the teleconnection pattern toward the marginal northwestern Pacific. Such wave energy propagation was possible since the modified Rossby wave theory considers the effect of basic zonal and meridional wind^19,24–26^ (Fig. 5a, Supplementary Fig. S4). According to the climatology, the meridional vorticity gradient showed a positive value across 40–60°N and 30–120°E (Fig. 5a), which was proportional to the polar front westerly^27,28^ and the northerly wind over Ural-Siberia (Supplementary Fig. S4). Along the westerly and northerly background flows that are included in the dispersion relationship for the barotropic Rossby wave, the stationary wave can penetrate southeastward into the East Sea.

This wave train pattern is analogous to the East Atlantic/Western Russia (EAWR) teleconnection, which is known as the dominant atmospheric teleconnection that affects Eurasian weather and climate during boreal winter^29–34^. The positive phase of the EAWR pattern has positive height anomalies over Europe and northern China and negative over the central North Atlantic and north of the Caspian Sea, and vice versa for the negative EAWR. External and internal forcing could generate and maintain atmospheric wave trains in mid-latitude^19,29,32,33,35,36^. As external forcing factor, anomalous boundary condition such as North Atlantic Sea surface temperature and North American/Eurasian snow cover could induce the upper-level divergence wind, acting as the Rossby wave source for the EAWR pattern^32,33,35^. Also, as internal dynamics, the interaction between background westerly and synoptic-scale transient eddies could drive the atmospheric Rossby wave energy propagation from the North Atlantic^29,33^. The spatial map of 200 hPa geopotential height anomalies was regressed against the EAWR index for 1982–2020 (Supplementary Fig. S5). This height regression field with the EAWR index showed a consistent pattern to the observation of the geopotential height anomaly in April 2020 (Fig. 2a). The EAWR index exhibited a strong positive correlation coefficient of 0.89 with the wave train index (defined the index in this study) for 39 years, suggesting that the EAWR pattern accounts for approximately 79% of the year-to-year variance of wave train patterns.

In the meridional direction, the barotropic high-pressure anomaly over Siberia appeared with the baroclinic low-pressure anomaly over the East Sea, similar to the observed blocking pattern (Fig. 4). The atmospheric blocking system is closely connected with the wind speed variation in the upper-level troposphere. The 200 hPa zonal winds showed a strong negative anomaly (i.e., easterly wind) over Mongolia–northeastern China in April 2020 compared with the climatological average but a strong positive anomaly over northern Russia and southern China (Fig. 5b). This wind structure of the positive﻿-negative-positive anomalies near 120°E corresponded to the anomalous anticyclone and cyclone over Siberia and the East Sea, respectively. Furthermore, the zonal wind anomaly exhibited a wave-like pattern over 40–80°N, resembling the distribution of blocking frequency anomaly (Fig. 5b, Supplementary Fig. S2c): anomalous easterly (westerly) wind region coincided with a reduced (increased) blocking area. This result indicates that the Siberian blocking is relevant to the Rossby wave train pattern over Eurasia^10,14,37,38^. As shown in Fig. 4a, the Rossby wave train transferred the wave energy from upstream of Europe to downstream of Asia; this convergence of wave activity flux can reinforce and rebuild the anticyclonic circulation over Siberia; thus, likely creating a more intense and persistent blocking.

## Summary and discussion

This study elucidates that the causes of the dipole atmospheric circulation over Siberia and the East Sea associated with the extreme cold in April 2020 throughout Northeast Asia. The anomalous ridge and trough structure were a mixed pattern of stationary Rossby wave train and blocking. The wave train with alternating anticyclonic and cyclonic circulations across Eurasia was induced by the vorticity forcing of northwestern/central Russia and propagated from Western Europe to the East Sea. The climatological polar front jet and northerly wind allowed the wave energy to propagate toward the southeast. This wave pattern almost corresponds to the EAWR teleconnection pattern, which is an influential atmospheric circulation that influences winter weather in East Asia. In addition, the blocking frequency increased by approximately eleven times over Siberia in April 2020 compared with the climatological average. This region of increased blocking days over Siberia nearly paralleled the easterly (westerly) wind anomaly in April 2020 over Mongolia–Northeast China (northern Russia). The change in blocking frequency distribution was related to the wavy zonal flow change across the high latitude, similar to the Rossby wave pattern. The Rossby wave response can reestablish and re-strengthen the blocking high^10,14,37,38^.

The reason that the Siberian blocking frequency in April 2020 was the strongest over the past 39 years remains still nebulous. Many previous studies demonstrated that the Siberian blocking tends to occur in stronger and longer cold condition over East Asia during negative AO periods in winter^11,12,39–42^. However, no relationship between blocking and AO was identified in April 2020; the AO index was positive (0.93), while the blocking index had the highest value of 11.6 days. Accompanied by the exceptional stratospheric polar vortex, the AO index was extraordinarily robust at 2.83 in the winter and early spring (January–March) of 2020 among the last four decades^43^. This record low-frequency variability enabled maintaining the pattern of the cold Arctic and warm Eurasia until March 2020 through the strengthened westerly flow. Then, the AO index became weaker in April and more neutral in May (−0.03) than during January–March, coinciding with the change in westerly circulation surrounding the Arctic in April (Fig. 5b). We suggest that the AO phase transition from an extreme positive value in January–March to a weak negative value in May (from 2.83 to −0.03) may lead to the blocking over Siberia. Further study is warranted to clarify the relationship between the blocking and AO phase transition in springtime.

Our study highlights that extreme Siberian blocking and strong EAWR pattern influenced the dipole atmospheric circulation causing the cold condition in Northeast Asia during April 2020. This cold condition seriously harmed agricultural and marine ecosystems, such as fruit tree blossoms, farm sprouts, and phytoplankton spring blooms^7^, during the peak growth period over northeastern Asia, resulting in great economic losses throughout this region (www.cma.gov.cn and www.mafra.go.kr). Therefore, the current results will provide a better understanding of the extreme weather or climate conditions over northeastern Asia in April and may aid in decreasing the associated damage to land–ocean ecosystems.

## Methods

### Reanalysis data

We used monthly and daily mean data for 39 years from 1982 to 2020 from ERA5^44^. The reanalysis data have a 0.25° × 0.25° longitude-latitude grid at 37 vertical pressure levels for the atmospheric variables. The 2020 anomalies for each variable were calculated by removing the climatology mean from 1982 to 2019. The indices used in this study are: the dipole atmospheric circulation index as the normalized geopotential height of 500 hPa anomalies averaged over Siberia (50–70°N, 80–120°E) minus the East Sea (25–40°N, 125–145°E), which is an important factor relevant to the April cold condition over Northeast Asia; the wave train index as the normalized geopotential height of 500 hPa anomalies averaged over Western/Central Europe (45–60°N, 5°W–15°E) minus northwestern/central Russia (50–70°N, 30–60°E); the blocking index as the sum of the total number of blocking frequencies per month over Siberia. The defined regions (Western/Central Europe, northwestern/central Russia, Siberia, and the East Sea; see Fig. 2b for its location) were based on the location of the maximum or minimum 200 and 500 hPa geopotential height anomalies in April 2020. We also used the EAWR and AO indices from the website of the National Oceanic and Atmospheric Administration Climate Prediction Center.

### Model configuration and experiment design

This study employed the linear baroclinic model LBM^45^ to investigate the steady-state atmospheric circulation response by the vorticity or diabatic forcing^19–21^. The LBM code is available from https://ccsr.aori.u-tokyo.ac.jp/~lbm/sub/lbm.html. The model with linearized primitive equations has a horizontal resolution of T42 and 20 vertical sigma levels, and it requires a basic state and steady forcing or initial perturbation. The numerical experiment was performed using the vertical profile of the vorticity tendency anomaly for April 2020 in the northwestern/central Russia region (Supplementary Fig. S3). This positive vorticity forcing, which was obtained from EAR5, was given as steady forcing in the experiment. The background state in the experiments was set to the April climatological field taken from ERA5, which includes geopotential height, relative humidity, specific humidity, temperature, three-dimensional winds, and sea level pressure.

### Ray tracing

We applied the ray tracing algorithm to analyze the pathway of Rossby wave energy propagation generated by the vorticity forcing^24–26,46,47^. The dispersion relationship for barotropic nondivergence wave in a non-uniform basic horizontal flow with a meridional wind component^24,25^ is presented as follows:

$$\omega ={\overline{u} }_{M}k+{\overline{v} }_{M}l+\frac{{\overline{q} }_{x}l-{\overline{q} }_{y}k}{{K}^{2}}$$

where ${\overline{q} }_{x}$ and ${\overline{q} }_{y}$ are the zonal and meridional gradients of the absolute vorticity, respectively. These parameters are expressed as follows:

$${\overline{q} }_{x}=\frac{1}{{a}^{2}cos\phi }\left(\frac{{\partial }^{2}\overline{v}}{\partial {\lambda }^{2}}-\frac{{\partial }^{2}\overline{u}}{\partial \lambda \partial \phi }cos\phi +\frac{\partial \overline{u}}{\partial \lambda }sin\phi \right)\; and\; {\overline{q} }_{y}= \overline{{\beta }_{M}}+\frac{{\partial }^{2}\overline{v}}{\partial \lambda \partial \phi }+tan\phi \frac{\partial \overline{v}}{\partial \lambda }$$

where ${\overline{\beta }}_{M}$ is the poleward absolute vorticity gradient of $\frac{\partial f}{\partial y}-\frac{{\partial }^{2}{\overline{u} }_{M}}{\partial {y}^{2}}$; ${\overline{u} }_{M}$ and ${\overline{v} }_{M}$ are the background zonal and meridional flow on a Mercator projection, respectively; $k$ and $l$ are the zonal and meridional wavenumber; $\omega$ is the frequency, where $\omega =0$ denotes the case of the stationary Rossby wave. A more detailed description of this dispersion relationship has been previously reported by Li et al.^24^ and Zhao et al.^25^.

### Blocking frequency

The hybrid blocking frequency detection is a suitable method for detecting atmospheric blocking using long-term datasets^15,16^. This blocking method combines the advantages of the most commonly used blocking indices, i.e., the Dole–Gordon index^17^ and the Tibaldi–Molteni index^18^. The hybrid approach can detect blocking systems accurately by considering a meridional gradient reversal to reduce the erroneous classification of blocking (such as excluding a misdetection of quasi-stationary high-pressure and including an omega-shaped blocking). To detect the event of hybrid blocking, we followed the four-step procedure described by Dunn-Sigouin et al.^15^. Step 1: The 500 hPa geopotential height anomaly was defined as ${Z}^{^{\prime}}=Z-\overline{Z }-\widehat{Z}$ ^48,49^, where $Z$ is the normalized geopotential height by the sine of latitude, $\overline{Z }$ is the running annual-mean of $Z$ centered on a given day, and $\widehat{Z}$ is the seasonal mean cycle derived from the running monthly mean of $Z-\overline{Z }$ centered on a given day. Step 2: The closed contour of the blocking anomaly (positive ${Z}^{^{\prime}}$) was identified using spatial criteria based on the minimum amplitude and spatial scale, and temporal criteria based on minimum overlap in the blocking area within two days. The minimum amplitude threshold was set to 1.5 standard deviation of ${Z}^{^{\prime}}$ over 30–90°N for an average of three months centered on a given month. The spatial scale threshold was 2.5 × 10^6^ km^2^, with a minimum overlap threshold of 50%. Step 3: The closed contour of the blocking anomaly should also satisfy the absolute meridional $Z$ gradient’s reversal. Step 4: If the conditions in steps 2 and 3 were ensured for five consecutive days, then the blocking anomaly was referred to as an atmospheric blocking high.

## Supplementary Information

Supplementary Information.

## Data Availability

All data are publicly available from the following sites: ERA5 monthly and daily data at https://cds.climate.copernicus.eu. EAWR and AO indices at https://www.cpc.ncep.noaa.gov/. Simulated LBM data is available from the corresponding author on reasonable request.
