# Supplementary material for: Causes for an extreme cold condition over Northeast Asia during April 2020
Source: Sci Rep. 2023 Feb 27;13:3315. doi: 10.1038/s41598-023-29934-w (PMC9971255; doi:10.1038/s41598-023-29934-w)
Supplement: Supplementary file 1 — Supplementary Information. [file 41598_2023_29934_MOESM1_ESM.docx]

**Supplementary Information**

**Causes for an extreme cold condition over Northeast Asia during April 2020**

Go-Un Kim^1^, Hyoeun Oh^1^, Yong Sun Kim^1,2^, Jun-Hyeok Son^1,3^, and Jin-Yong Jeong^1^*‬‬‬‬‬‬‬‬‬‬‬‬‬‬‬‬‬‬

^1^Korea Institute of Ocean Science and Technology, Busan, South Korea

^2^Ocean Science and Technology School, Korea Maritime and Ocean University, Busan, South Korea

^3^Korea Power Exchange, Naju, South Korea

*Corresponding author: Dr. Jin-Yong Jeong (jyjeong@kiost.ac.kr)


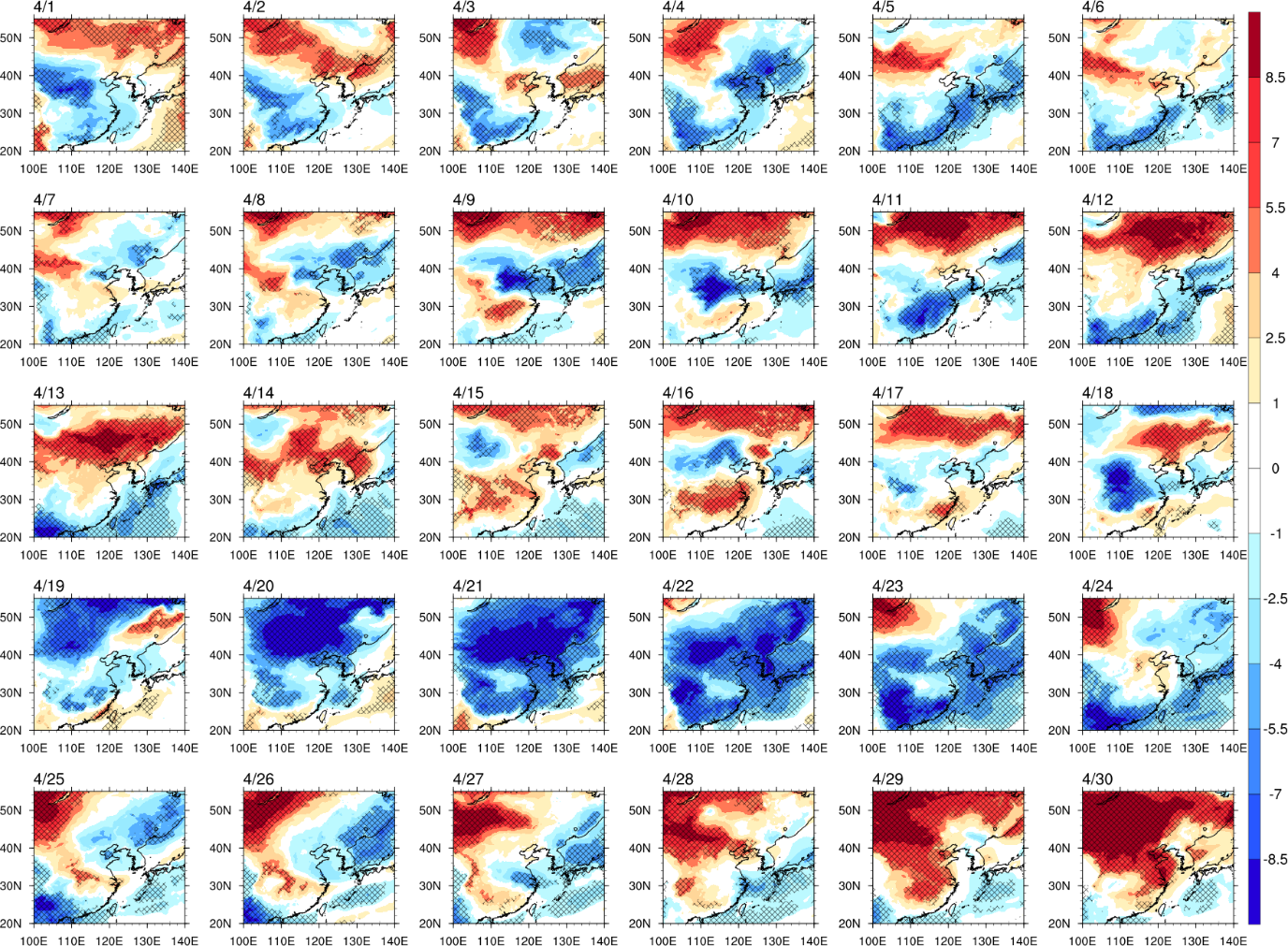


**Supplementary Figure S1. Daily temperature maps for April 2020.** Same as Fig. 1a but showing the period of 1–30 April, 2020 in ERA5. The maps were created using the NCAR Command Language Version 6.4.0 ([http://www.ncl.ucar.edu](http://www.ncl.ucar.edu/)).


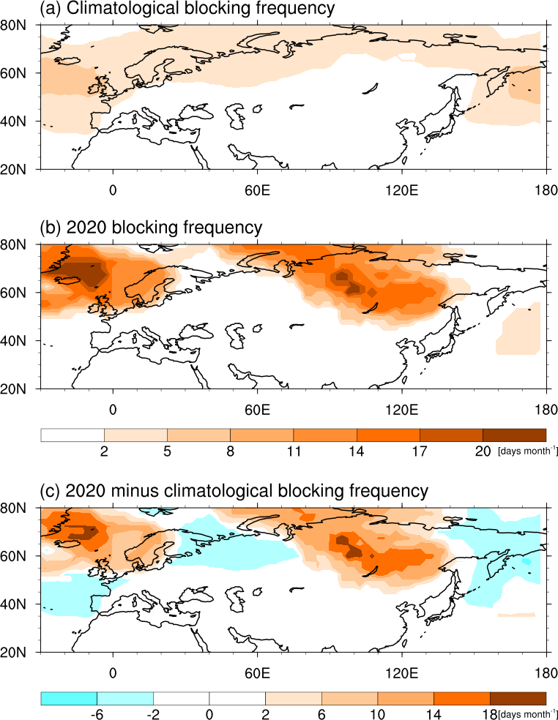


**Supplementary Figure S2. Distribution of blocking occurrence.** Total number of blocking frequency (shading, days per month) in April (a) climatological period 1982–2019, (b) 2020, and (c) 2020 minus climatology from ERA5. Maps were generated using the NCAR Command Language Version 6.4.0 ([http://www.ncl.ucar.edu](http://www.ncl.ucar.edu/)).


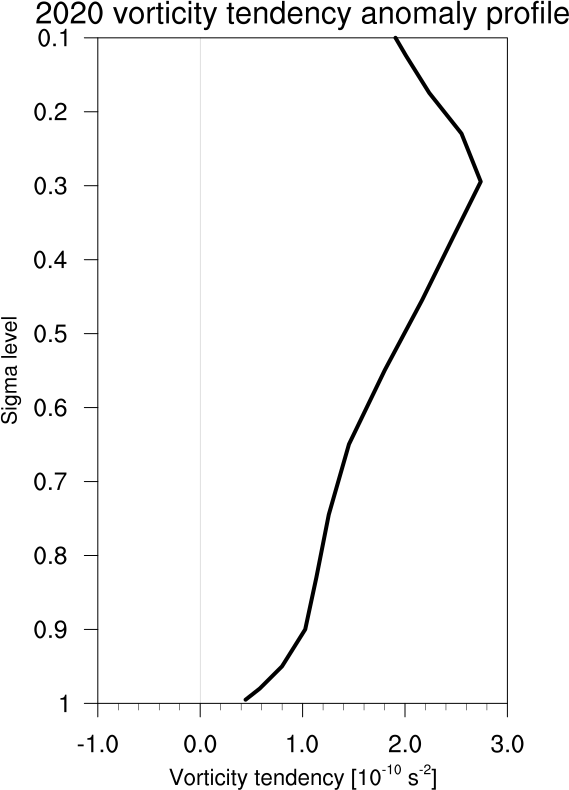


**Supplementary Figure S3. Vertical forcing profile.** Vertical profile of vorticity tendency anomaly averaged over the northwestern/central Russia region (50–70 °N, 30–60 °E) in April 2020.


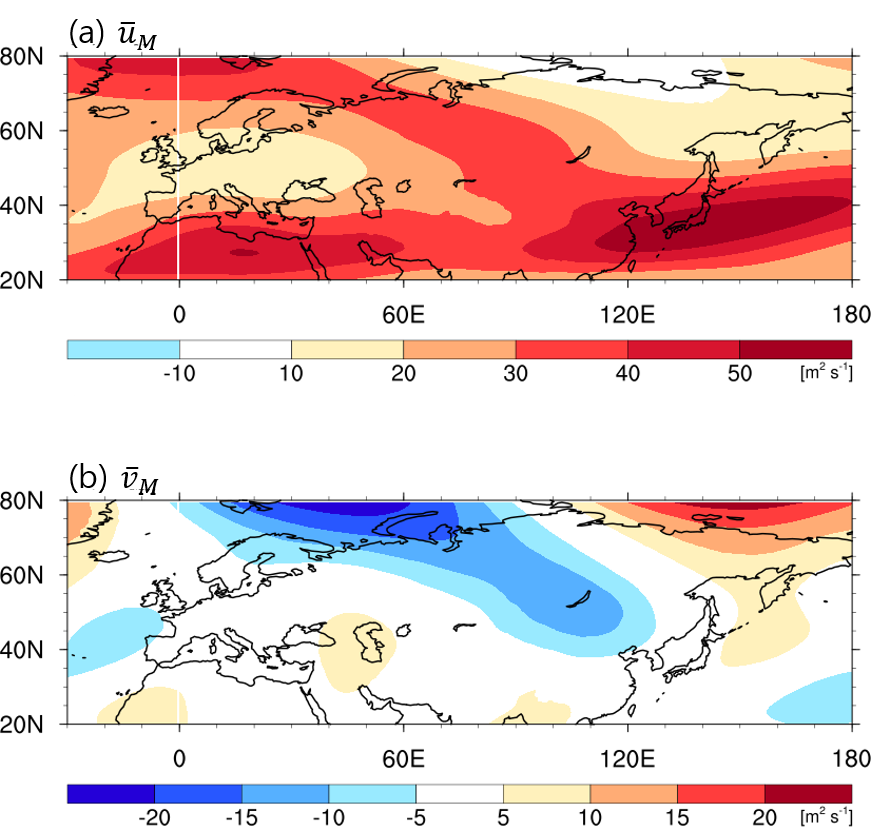


**Supplementary Figure S4. Potential causes for the cold condition.** Same as Fig. 5a but for the (a) 200 hPa zonal wind ($\bar{u}_{M}$, m s^-1^), and (b) 200 hPa meridional wind ($\bar{v}_{M}$, m s^-1^) in the Mercator coordinate. The maps were created using the NCAR Command Language Version 6.4.0 ([http://www.ncl.ucar.edu](http://www.ncl.ucar.edu/)).

**
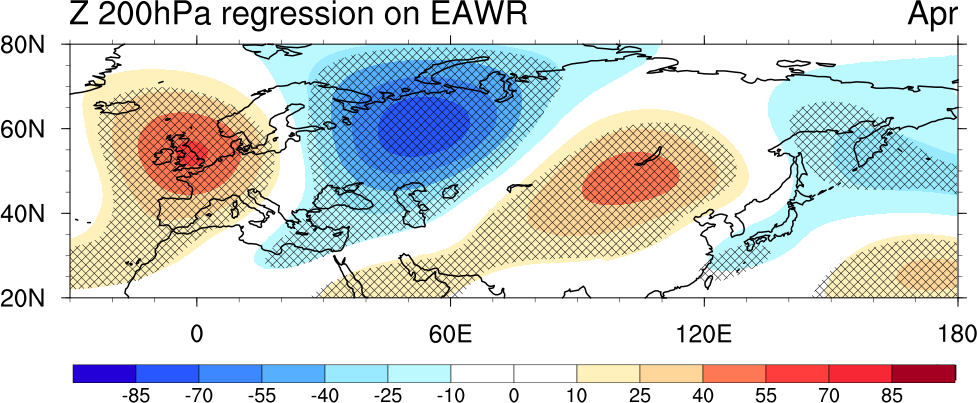
**

**Supplementary Figure S5. Regression pattern associated with the East Atlantic/Western Russia (EAWR) index.** Regressed field of the 200 hPa geopotential height anomalies against the EAWR index in April during 1982–2020. Hatched areas indicate the significant regions at the 90% confidence level. Map was generated using the NCAR Command Language Version 6.4.0 ([http://www.ncl.ucar.edu](http://www.ncl.ucar.edu/)).
